# Editorial: Leveraging multi-omics approaches to understand and manage gastrointestinal and hepatic diseases

**DOI:** 10.3389/fphar.2024.1529088

**Published:** 2024-12-13

**Authors:** Isis Trujillo-Gonzalez, Paul G. Thomes, Isin Tuna Sakallioglu, Kusum K. Kharbanda

**Affiliations:** ^1^ Department of Nutrition, University of North Carolina at Chapel Hill, Chapel Hill, NC, United States; ^2^ Nutrition Research Institute, University of North Carolina at Chapel Hill, Chapel Hill, NC, United States; ^3^ Department of Anatomy, Physiology, and Pharmacology, College of Veterinary Medicine, Auburn University, Auburn, AL, United States; ^4^ Department of Chemistry, University of Nebraska-Lincoln, Lincoln, NE, United States; ^5^ Department of Internal Medicine, University of Nebraska Medical Center, Omaha, NE, United States; ^6^ Department of Biochemistry and Molecular Biology, University of Nebraska Medical Center, Omaha, NE, United States; ^7^ Research Service, Veterans Affairs Nebraska-Western Iowa Healthcare System, Omaha, NE, United States

**Keywords:** metabolic dysfunction-associated steatotic liver disease, traditional Chinese medicine, cholangitis, gut micriobiome, inflammation

The Research Topic “Leveraging multi-omics approaches to understand and manage gastrointestinal and hepatic diseases” proposed by the Frontiers in Pharmacology has attracted a total of four articles on preclinical studies that were covered in three original research manuscripts and one review article. Two research articles were on metabolic dysfunction-associated steatotic liver disease (MASLD), previously named non-alcoholic fatty liver disease (NAFLD), which is now recognized as a predominant cause of chronic liver disease worldwide, affecting over 30% of the adult population (Teng et al., 2023; Miao et al., 2024). Current pharmacological treatments for MASLD are limited and, until a recent FDA-approved treatment, primarily addressed underlying conditions such as obesity, resulting in a non-standardized therapeutic approach predominantly focused on weight management. This scenario highlights the need for high-throughput, comprehensive alternative strategies to effectively treat MASLD, encompassing basic mechanistic research, pathway analysis, and the identification of novel drug targets. Additionally, advanced technologies, including untargeted omics methods and approaches that consider inter-organ communication, are essential for advancing therapeutic strategies for this complex disease.

In this Research Topic, Deng et al. explore the therapeutic potential of *Dendrobium huoshanense*, an edible medicinal plant known for its anti-inflammatory and antioxidative properties, in a mouse model of MASLD induced by a high-fat, high-fructose (HFHF) diet. Their findings reveal that *Dendrobium huoshanense* polysaccharide (DHP) significantly alleviates MASLD symptoms, notably by reducing lipid accumulation and inflammation. DHP treatment notably attenuates inflammation by inhibiting the Toll-like receptor 4 (TLR4) and NF-κB inflammatory pathways, with a concomitant reduction in multiple pro-inflammatory cytokines. Furthermore, an untargeted metabolomics approach identified 49 metabolites that were differentially regulated in the model group compared to DHP-treated groups. Notably, key metabolites such as niacinamide, choline, betaine, and glycine showed increased levels in response to DHP treatment, suggesting potential pathways for therapeutic exploration. Further research is necessary to elucidate the precise molecular mechanisms by which DHP modulates inflammation and to investigate how these mechanisms, in conjunction with specific metabolites, could be harnessed to develop more comprehensive anti-inflammatory therapies for MASLD.

There is growing interest in understanding the interplay between the gut microbiome and liver in the context of MASLD. A preclinical study by Chen et al. investigates this relationship using Shening Baizhu San (SBSZ), a traditional Chinese medicinal formula, to assess its impact on MASLD in mice consuming either a Western diet or a normal diet, with or without CCl_4_ injection. Their findings indicate that SBSZ treatment significantly lowered blood glucose, liver weight, hepatic triglycerides, and total cholesterol. Histological analysis further confirmed reduced lipid accumulation and inflammation, with mRNA expression levels of IL-10, IL-6, and TNF indicating an anti-inflammatory effect of SBSZ. The authors also examined the gut barrier, focusing on the serotonin pathway in the small intestine. They found that SBSZ reduced serotonin levels and decreased expression of the 5-HT receptor, suggesting a possible reduction in liver inflammation through this pathway. Although these results are promising, more detailed analyses are needed to fully understand the mechanism. Moving further, these authors analyzed gut microbiome composition and metabolite profiles, observing that SBSZ increased levels of beneficial gut bacteria such as *Bifidobacterium* and *Parvibacter*. Metabolomic analysis demonstrated that SBSZ normalized levels of key metabolites, including bile acids and tryptophan-related amino acids. Correlation analysis revealed that beneficial gut bacteria were associated with reduced liver inflammation and steatosis, while pathogenic species were positively correlated with indicators of MASLD severity. Future research is essential to evaluate the safety and efficacy of SBSZ in human subjects, as it has yet to receive FDA approval.

Chronic liver diseases characterized by autoimmune etiologies, such as primary sclerosing cholangitis (PSC) and primary biliary cholangitis (PBC), involve progressive injury and inflammation of the bile ducts, leading to complex chronic liver conditions. In their study, Pham et al. employed the deconvolution tool CIBERSORTx to estimate the cellular composition of liver tissue in cases of PSC and PBC. This study pioneers the use of custom signature matrices derived from single-cell RNA sequencing (scRNA-seq) data, enabling a more accurate capture of the complex cellular heterogeneity within hepatic tissues. The authors generated these signature matrices from an existing scRNA-seq database, allowing for the precise deconvolution of hepatic cell types, including stellate cells, hepatocytes, immune cells, and cholangiocytes.

To validate CIBERSORTx, the authors compared its results to known cellular distributions, assessing the tool’s accuracy in identifying cell types within liver tissue. Furthermore, they conducted validation using gene expression markers—such as COL1A1 for myofibroblasts—demonstrating not only cellular characteristics but also specific gene expression profiles, underscoring the model’s robustness. This research establishes that custom signature matrices allow CIBERSORTx to deconvolve liver tissue data with high specificity and accuracy. Further studies are warranted to explore the potential of this tool in analyzing cellular heterogeneity in metabolic-associated fatty liver diseases and other complex conditions.

Finally, in a review, Li et al. discusses the therapeutic potential of Traditional Chinese Medicine (TCM) compounds for treating hepatic fibrosis, with a particular focus on targeting angiogenesis. The paper discusses the efficacy of various compounds known for their anti-fibrotic, anti-inflammatory, and antioxidant properties, each contributing to the inhibition of pathological blood vessel formation. Among the most widely studied compounds are curcumin—a polyphenolic compound derived from turmeric that modulates angiogenic pathways; procyanidin B2—a flavonoid found in berries that disrupts the Hedgehog pathway; and salvianolic acid B, which prevents pathological angiogenesis by inhibiting VEGF signaling, among other TCM agents.

This study highlights the crucial role of hepatic stellate cells, which are activated during chronic liver injury and drive collagen accumulation, contributing to fibrosis progression. This review emphasizes the need for further investigation to validate these mechanisms and evaluate the safety and efficacy of TCM compounds in clinical settings, suggesting that TCM holds promise for developing novel therapeutic strategies in chronic liver disease management.

Overall, this Research Topic examines preclinical data, gut microbiome crosstalk, novel software approaches and discuss compounds that can uncover important pathways in liver diseases.

## References

[B1] Miao L. Targher G. Byrne C. D. Cao Y.-Y. Zheng M.-H. (2024). Current status and future trends of the global burden of MASLD. Trends Endocrinol. Metabolism 35 (Issue 8), 697–707. 10.1016/j.tem.2024.02.007 38429161

[B2] Teng M. L. Ng C. H. Huang D. Q. Chan K. E. Tan D. J. Lim W. H. (2023). Global incidence and prevalence of nonalcoholic fatty liver disease. Clin. Mol. Hepatol. 29 (Suppl. l), S32–S42. 10.3350/cmh.2022.0365 36517002 PMC10029957

